# The association of headache frequency with pain interference and the burden of disease is mediated by depression and sleep quality, but not anxiety, in chronic tension type headache

**DOI:** 10.1186/s10194-017-0730-5

**Published:** 2017-02-10

**Authors:** María Palacios-Ceña, Juan J. Fernández-Muñoz, Matteo Castaldo, Kelun Wang, Ángel Guerrero-Peral, Lars Arendt-Nielsen, César Fernández-de-las-Peñas

**Affiliations:** 1 Department Physical Therapy, Occupational Therapy, Rehabilitation, and Physical Medicine, University Rey Juan Carlos, Alcorcón, Spain; 2 Center for Sensory-Motor Interaction (SMI), Department of Health Science and Technology, School of Medicine, Aalborg University, Aalborg, Denmark; 3 Department of Psychology, Universidad Rey Juan Carlos, Alcorcón, Spain; 4 Master in Sport Physiotherapy, University of Siena, Siena, Italy; 5 Poliambulatorio Fisiocenter, Collecchio, Parma, Italy; 6 Headache Unit. Hospital Clínico Universitario de Valladolid, Valladolid, Spain

**Keywords:** Tension type headache, Sleep quality, Depression, Anxiety

## Abstract

**Background:**

A better understanding of potential relationship between mood disorders, sleep quality, pain, and headache frequency may assist clinicians in determining optimal therapeutic programs. The aim of the current study was to analyze the effects of sleep quality, anxiety, depression on potential relationships between headache intensity, burden of headache, and headache frequency in chronic tension type headache (CTTH).

**Methods:**

One hundred and ninety-three individuals with CTTH participated. Headache features were collected with a 4-weeks headache diary. The Hospital Anxiety and Depression Scale was used for assessing anxiety and depression. Headache Disability Inventory evaluated the burden of headache. Pain interference was determined with the bodily pain domain (SF-36 questionnaire). Sleep quality was assessed with Pittsburgh Sleep Quality Index. Path analyses with maximum likelihood estimations were conducted to determine the direct and indirect effects of depression, anxiety, and sleep quality on the frequency of headaches.

**Result:**

Two paths were observed: the first with depression and the second with sleep quality as mediators. Direct effects were noted from sleep quality, emotional burden of disease and pain interference on depression, and from depression to headache frequency. The first path showed indirect effects of depression from emotional burden and from sleep quality to headache frequency (first model *R* ^2^ = 0.12). Direct effects from the second path were from depression and pain interference on sleep quality and from sleep quality on headache frequency. Sleep quality indirectly mediated the effects of depression, emotional burden and pain interference on headache frequency (second model *R* ^2^ = 0.18).

**Conclusions:**

Depression and sleep quality, but not anxiety, mediated the relationship between headache frequency and the emotional burden of disease and pain interference in CTTH.

## Background

Tension type headache is a common headache disorder with a global prevalence of 42% [1] and has a large socio-economic impact [2]. The general costs in Europe in 2010 for primary headaches were €13.8 billion [3]. In fact, in the Global Burden of Disease Study, it was found that tension type headache was the second most prevalent disorder in the world [4].

Current research regarding the pathogenesis of tension type headache is focused on altered nociceptive pain processing and its role on chronification [5]. It appears that among the clinical features of headache, the frequency of attacks is the most relevant outcome since higher frequency of attacks is associated with higher sensitization [6]. In addition, emotional factors, such as depression or anxiety, and poor sleep quality can also play a role in the sensitization process by increasing excitability of the nociceptive firing [7, 8]. Chiu et al. reported that poor sleep quality and depression were independently associated with reduced pain thresholds, supporting this assumption [9].

There is evidence suggesting that subjects with tension type headache exhibit co-morbid anxiety, depression [10] and sleep insomnia [11]. Interestingly, depression and anxiety are more associated to the headache frequency than to headache diagnosis [12]. Further, poor sleep quality is usually considered a risk factor for progression from the episodic to the chronic form of tension type headache [13]. It seems that the severity and the prevalence of sleep problems increase proportionally to the frequency of the headaches [14]. It is therefore conceivable that both depression and sleep quality can interact at different levels by promoting higher frequency of headaches. Additionally, individuals with tension type headache and comorbid psychiatric disorders often exhibit affective temperament dys-regulation and suicidal behaviors, which may also contribute to this chronification process [15].

In fact, poor sleep quality has been related to higher levels of depression and pain intensity [16] and lower functioning [17] in patients with chronic pain. A better understanding of the potential relationship between mood disorders, i.e., depression and anxiety, and sleep quality with the frequency of headaches in individuals with tension type headache could assist clinicians for determining better therapeutic programs and hence for preventing evolution to the chronic form of the disease. However, no study has investigated potential direct and indirect effects of these variables with headache frequency at the same time in this headache condition. Since depression and sleep disturbances are associated with the frequency of headaches, we included individuals with chronic tension type headache (CTTH). Therefore, the purpose of the current study was to determine the direct and indirect effects of sleep quality, anxiety or depression on the potential relationships between headache intensity, burden of the condition, pain interference and headache frequency in individuals with CTTH. We hypothesized that mood disorders, i.e., depression and anxiety, and poor sleep quality would mediate the association between headache and related-disability with the frequency of headaches.

## Methods

### Participants

Consecutive patients with a diagnosis of tension type headache were recruited from different university-based hospitals from September 2014 to July 2016. Patients were recruited during routine medical visit. Diagnosis was conducted according to the criteria of the International Classification of Headache Disorders, third edition (ICHD-III beta, 2013) down to third-digit level (code 2.3) by an expert neurologist in the diagnosis and management of headaches [18]. To be included, patients had to describe all the pain features of TTH: bilateral location, pressing and tightening pain, moderate intensity (≤6 on a 10-points numerical pain rate scale, NPRS) and no aggravation of pain during physical activity. Patients should also report neither more than one of photophobia, phonophobia or mild nausea and neither moderate nor severe nausea nor vomiting as requested by the ICHD-III diagnostic criteria [18]. Headache attacks had to be present from at least 3 months with a frequency higher than 15 days per month [18].

Participants were excluded if they presented: 1, patients with epidosic headaches; 2, other primary/secondary headaches including medication overuse headache as defined by the ICHD-III; 3, history of neck or head trauma (i.e., whiplash injury); 4, systemic degenerative diseases, e.g., rheumatoid arthritis, lupus erythematous; 5, diagnosis of fibromyalgia syndrome; 6, receiving anesthetic blocks within the previous 6 months; 7, physical treatment in the neck and/or head received the previous 6 months; 8, abuse of caffeine or other stimulating substances; or, 9, pregnancy.

All participants read and signed a consent form prior to their participation. The local Ethics Committee approved the study (URJC 23/2014, HUFA 14/104, Aalborg N20140063, CESU 5/2015) which took place at three countries (Spain, Denmark, Italy).

### Headache clinical outcomes - headache diary

A headache diary over 4 weeks was used to substantiate the diagnosis and to record the headache clinical features [19, 20]. On this diary, patients registered the frequency of headaches (days per week), the headache intensity on an 11points numerical pain rate scale [21] (NPRS; 0: no pain, 10: the maximum pain), and the duration of each headache attack (hours per day). The main outcome in this study was the frequency of headaches.

### Hospital Anxiety and Depression Scale (HADS)

The HADS is a 14 item self-report screening scale, seven items for anxiety (HADS-A) and seven for depression (HADS-D), developed to indicate the presence of anxiety and depressive symptoms [22]. Each item scores on a Likert scale (0–3) giving a maximum subscale score of 21 points for each subscale [23]. The HADS has shown good validity and internal consistency (Cronbach's α: 0.84) for being used in subjects with headache [24].

### Sleep quality

Sleep quality was assessed with the Pittsburgh Sleep Quality Index (PSQI) [25]. This questionnaire assesses sleep quality over a 1-month period by including 19 self-rated questions and five questions answered by bedmates or roommates. Items use varying response categories recording usual bed time, usual wake time, number of actual hours slept, and number of minutes to fall asleep. All questions are answered on a Likert-type scale (0–3). The sum of all answered for the components yields one global score (0–21) where higher score indicates worse sleep quality. Buysse et al. reported that the PSQI has good internal consistency (α: 0.83) and test-retest reliability (r: 0.85) [26]. A total score > 8.0 has been found to be indicative of poor sleep quality [27].

### Headache Disability Inventory (HDI)

The HDI assesses the burden of headache using 25 items that inquire about the perceived impact of headache on emotional functioning and daily life activities [28]. Possible answers for each item are YES (4 points), SOMETIMES (2 points) and NO (0 points). Thirteen items asses the emotional component of headache (HDI-E, maximum score: 52) and the remaining 12 items assess the physical component (HDI-P, maximum score: 48). A higher score suggests a greater burden of headache for each subscale. The HDI has exhibited good stability at short (*r* = 0.93-0.95) and long (*r* = 0.76-0.83) term follow-ups [29].

### Pain interference

To determine the interference of pain, we used the bodily pain domain of the health-related quality of life Medical Outcomes Study Short Form 36 (SF-36) questionnaire [30]. After summing the Likert-scaled items of this domain, it is categorized from 0 (lowest level of pain) to 100 (highest level of pain) [31]. Lower scores represent higher interference of pain.

### Statistical analysis

Means and confidence intervals were calculated to describe the outcomes. The Kolmogorov-Smirnov test revealed that all data had a normal distribution (*P* > 0.05). To determine the relationship between the dependent measure (the frequency of headaches) and the independent outcomes (headache intensity, headache duration, HDI-E, HDI-P, sleep quality, pain interference, HADS-D and HADS-A), different Pearson product–moment correlation coefficients were first assessed.

Since anxiety was not significantly associated with the frequency of headaches, we conducted two different path analyses with depression and sleep quality as independent measures, since both were associated with headache frequency. Two path analyses with maximum likelihood estimations were conducted to evaluate the potential direct and indirect effects of independent variables on the association between headache frequency with headache intensity, headache duration, sleep quality, HDI-E, HDI-P, interference of pain, and HADS-D using AMOS computer program [32]. A path model analysis is a regression model extension relating independent, intermediary and dependent variables [33]. In the hypothesized model, the frequency of headache was the dependent variable; headache intensity, headache duration, HDI-E, HDI-P, and interference of pain were the independent outcomes, and sleep quality and depression (HADS-D) were intermediary variables. In a path analysis, single arrows indicate causation between intermediary and dependent variable. Further, arrows also connect the error terms with their respective intermediary variables. Double arrows indicate correlation between pairs of independent variables. The path coefficient is a standardized regression coefficient (beta) showing the direct effect of an independent variable (pain interference) on a dependent (headache frequency) variable. These path coefficients may be used to decompose correlations into direct and indirect effects, corresponding to direct and indirect paths reflected within the arrows in the model. Indirect effects occur when the relationship between two variables (e.g. headache frequency and interference of pain) is mediated by one or more variables (i.e., depression or sleep quality).

In the first model, headache intensity, sleep, HDI-E, HDI-P and pain interference were identified as predictors of depression. Likewise, depression was specified as a predictor of headache frequency. In the second model, headache intensity, HDI-E, HDI-P, HADS-D and pain interference were identified as predictors of sleep quality, and sleep quality was also specified as predictor of the frequency of headaches.

The evaluation of each model data was based on several recommended indexes. AMOS provides several fit of them that are largely independent of the sample size: chi-square statistic (*X* ^*2*^) [34, 35]; the goodness of fit index (GFI) and adjusted goodness of fin index (AGFI) whose value reference is at 90 to consider an acceptable model [36], and the comparative fin index (CFI) which accepted adequate value is over 0.90 [37]. Finally, within parsimony adjustment indices, the errors of the root mean square approximation (RMSEA) whose values < 0.08 are good to accept the model [38] were also calculated. Missing data were treated with maximum likelihood imputation.

## Results

### Clinical data of the sample

A total of 250 individuals with headache were screened for possible eligibility criteria. Finally, 193 (73% women) satisfied all eligibility criteria of CTTH, agreed to participate and signed the informed consent. The remaining 57 were excluded for the following reasons: co-morbid migraine (*n* = 27), episodic tension type headache (*n* = 10), previous whiplash (*n* = 10), fibromyalgia (*n* = 5) and medication overuse headache (*n* = 5). Seventy-five (38%) of the patients were taking prophylactic drugs (i.e., amitriptyline) on a regular basis. Demographic data and outcome measure scores are listed in Table 1.

**Table 1:** Demographic variables and pearson-product moment correlation matrix for each study variable

	Mean	95% CI	1	2	3	4	5	6	7	8
1. Headache frequency (days/month)	18.1	17.5–18.7								
2. Headache intensity (0–10)	5.9	5.5–6.3	.145^*^							
3. Headache duration (hours/attack)	7.3	6.6-8.0	.249**	n.s						
4. Pittsburg Questionnaire (0–21)	17.1	15.8–18.4	.223^**^	.177^**^	.165*					
5. HDI-E (0–52)	18.7	17.0–20.4	.341^**^	.212^**^	.350**	.328^**^				
6. HDI-P (0–48)	22.5	20.9–24.1	.235^**^	.165^*^	.237**	.232^**^	.828^**^			
7. Bodily Pain (0–100)	51.2	47.9–54.6	-.245^**^	-.165^*^	n.s.	-.350^**^	-.409^**^	-.442^**^		
8. HADS-D (0–21)	8.0	7.5–8.6	.274^**^	.227^**^	.313**	.426^**^	.538^**^	.405^**^	-.389^**^	
9. HADS-A (0–21)	10.0	9.3-10.7	n.s.	n.s	n.s	n.s	n.s	n.s	n.s	.208**

95% CI: 95% confidence interval

*HDI* Headache Disability Inventory (*E* Emotional, *P* Physical), *HADS* Hospital Anxiety and Depression Scale (*D* Depression, *A* Anxiety)

*P < 0.05; **P < 0.01

### Correlation analysis

Table 1 summarizes the Pearson’s correlation coefficients and the descriptive analysis between all variables. Significant positive correlations were found between the frequency of headaches with headache intensity, headache duration, sleep quality, HDI-E, HDI-P and HADS-D: the greater the headache intensity, the longer the headache duration, the worse the quality of sleep, the higher the emotional or physical component of the headache and the higher the depression, the higher the frequency of headache attacks. Further, headache frequency was negatively associated with pain interference: the lower the bodily pain score, i.e., the higher the inference of pain, the higher the frequency of headaches.

### First path analysis - depression as mediator

**Fig. 1:**
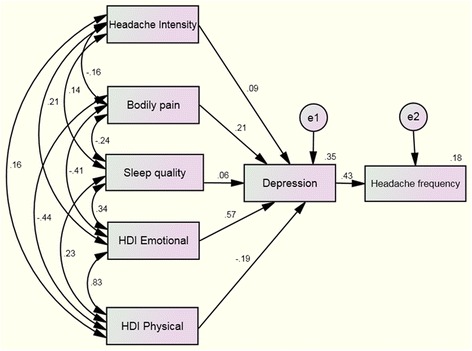
Path analyses to headache frequency from headache intensity, sleep quality, bodily pain, and burden of headache (physical and emotional) with depression as the mediating variable. Standardized direct path coefficients are presented. Data from *double arrows* represent correlations between the variables whereas data from *single arrows* represent standardized regression weights (parameter estimates). e1 and e2: error terms for each variable

The hypothesized model fit the data was excellent, with X^2^ = 3.14 X^2^/df = 5, Goodness of Fit Index (GFI): 0.96; Adjusted Goodness of Fit Index (AGFI): 0.91; Comparative Fit Index (CFI): 0.97, and Normal Fit Index (NFI): 0.97. Further, Root Mean Square Error of Approximation (RMSEA) was 0.07. Figure 1 displays all the parameter estimates (standardized solution).

According to the direct effects, significant paths were noted from sleep quality (B = 0.25; *P* < 0.01), HDI-E (B = 0.16; *P* < 0.01) and pain interference (B = −0.04; *P* < 0.05) on depression. Likewise, a significant path was also indicated from depression (B = 0.71; *P* < 0.01) on headache frequency. The direct effects from HDI-P (B = 0.15; *P* = 0.133) and headache intensity (B = 0.15; *P* = 0.166) on depression were not significant. The amount of depression explained by all predictors in this model was *R* ^2^ = 0.42.

Further, the path analysis included an indirect effect from HDI-E to headache frequency, exerted through depression (B = 0.13, *P* < 0.01), and from the sleep quality to headache frequency, also exerted through depression (B = 0.06, *P* < 0.01). The indirect effect of depression on the remaining outcomes was not statistically related to headache frequency: pain interference (B = −0.04, *P* > 0.05), headache intensity (B = 0.02, *P* > 0.05), and HDI-P (B = −0.04, *P* > 0.05). Overall, the amount of headache frequency explained by all predictors in this first model was *R* ^2^ = 0.12.

### Second path analysis - sleep quality as mediator

**Fig. 2:**
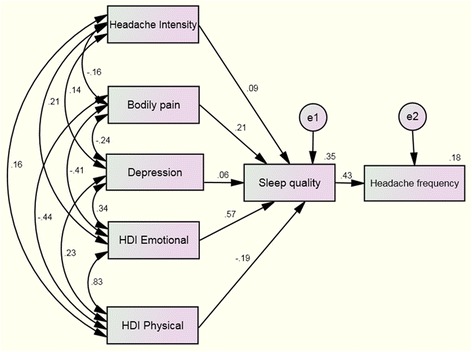
Path analyses to headache frequency from headache intensity, depression, bodily pain, and burden of headache (physical and emotional) with sleep quality as the mediating variable. Standardized direct path coefficients are presented. Data from *double arrows* represent correlations between the variables, where data from *single arrows* represents the standardized regression weight (parameter estimates). e1 and e2: error terms for each variable

The hypothesized model fit the data was also excellent, with X^2^ = 4.42 X^2^/df = 5, Goodness of Fit Index (GFI): 0.97; Adjusted Goodness of Fit Index (AGFI): 0.84; Comparative Fit Index (CFI): 0.96, and Normal Fit Index (NFI): 0.96. In addition, Root Mean Square Error of Approximation (RMSEA) was 0.07. Figure 2 displays all the parameter estimates (standardized solution).

According to the direct effects, significant paths were observed from depression (B = 0.29; *P* < 0.01) and pain interference (B = −0.03; *P* < 0.01) on sleep quality. A significant path was also indicated from sleep quality (B = 0.48; *P* < 0.01) on headache frequency. The rest direct effects were not significant. The amount of sleep quality explained by predictors in this model was *R* ^2^ = 0.42.

The path analysis model also included several indirect effects of sleep quality from depression (B = 0.06; *P* < 0.01), HDI-E (B = 0.04; *P* < 0.01) and pain interference (B = −0.04; *P* < 0.01) on headache frequency. The indirect effects of sleep quality on the remaining predictors were not significantly related to the frequency of headache attacks: headache intensity (B = 0.01; *P* > 0.05) and HDI-P (B = −0.02; *P* > 0.05). Overall, the amount of headache frequency explained by all predictors in this second model was *R* ^2^ = 0.18.

## Discussion

This is the first study investigating the indirect effects of depression, anxiety and sleep quality on headache frequency in individuals with tension type headache. We observed that depression and sleep quality, but not anxiety, mediated the relationship between the frequency of headaches with the emotional burden of condition and pain interference in subjects with CTTH. These results support the assumption that headache frequency is related to the emotional burden of the condition but with an indirect mediator effect of depression and sleep quality; whereas the relation with interference of pain is only mediated by sleep quality.

The findings from this study show, firstly, and in accordance with prior literature, that depression and sleep quality are factors associated with the frequency of headaches in CTTH [12, 14]. The study also demonstrated that depressive levels and sleep quality mediated the relationship between the emotional burden of headache and the frequency of attacks. Thus, while a small amount of variance (12% or 18%) within the relationship between the emotional burden of condition and headache frequency was indirectly mediated by depression or sleep quality, respectively, the relationships with other variables were also significant (although attenuated). This suggests that the emotional burden of headache may contribute to the frequency of headaches via other factors. For instance, emotional stress is associated with increased mechanical pain hypersensitivity [7]; therefore, it is possible that a higher emotional burden of headache (stressful situation) can contribute to the excitability of the central nervous system and therefore increasing the frequency of headache attacks. Similarly, poor sleep quality and depression are also associated with increased sensitivity [9]; therefore, a synergistic effect may occur in headache sufferers who do not sleep well under stressful emotional situations. In fact, sleep deficiency is associated with more severe headaches [39] which would explain why sleep quality, but not depression, also mediated the relationship between the pain interference (i.e., bodily pain domain) and headache frequency. Finally, other aspects not assessed in the current study may also be involved in these interactions.

Depression and anxiety have a significant impact on quality of life and increase the burden on patients with headache [40]; however in the current study depression, but not anxiety, was found to have a significant indirect effect on headache frequency. Although current methodology does not allow determining the mechanisms involved in the mediating relationship of depression on headache frequency; it has been previously suggested that depression predominantly contributes to chronic pain via supra-spinal mechanisms and emotional modulation of pain [41].

The indirect effect of sleep quality on the frequency of headaches was slightly higher than depression, which agrees with previous research supporting a correlation between sleep quality and the frequency of headaches [13]. In addition, in the second path revealed that sleep quality also mediated the relationship between pain interference and the frequency of headaches, suggesting that different indirect effects are involved on each model. However, whether poor sleep quality leads to headache or conversely is unclear since underlying mechanisms of both conditions are shared by common pathogenic mechanisms [13].

Uncertainty over biological mechanisms in these interactions exists, however our results have clinical implications. Since emotional stress and poor sleep quality are the most common triggers for tension type headache [42, 43], proper management of their associated factors seems to be relevant. In fact, emotional stress, depression, and sleep are of clinical interest because they represent modifiable risk factors implicated in the chronicity of headaches [44]. Our study found an interaction between depression, the emotional burden of headache, sleep quality and frequency of headaches in subjects with CTTH. Therefore, proper copying management of stressful emotional events per se may not be the primary mechanism of action for decreasing the frequency of headache attacks in CTTH. Rather, the mechanism may be a reduction of depressive symptoms, or an alteration in the relationship between sleep quality and these emotional factors. In fact, current findings would suggest that management of patients with CTTH should include therapeutic interventions targeted to decrease the emotional burden of headache (copying strategies or cognitive behavioral techniques), to decrease depressive symptoms (i.e., psychological approaches) and to improve sleep quality.

Although strengths of this study include a large sample size and the inclusion of CTTH patients according to the most updated diagnostic criteria, the use of diagnostic diaries and the use of standardized instruments; our study has its limitation due to the fact that we included a sample consisted mainly of CTTH patients referred to a tertiary headache center and thus not representative of the general population. Additionally, the impact of daily medications to prevent headache was not considered in the path models. However, since 38% of our sample was taking preventive drugs and because headache history and headache features were comparable to previous studies, our study sample was probably fairly representative for a patient group encountered in a clinical setting. Second, the study is cross-sectional and causal relations are thus impossible to ascertain. Third, it should be noted that the HADS is a screening rather than diagnostic instrument for depression and anxiety symptoms with a tendency to underestimate prevalence of both disorders [45]. In fact, we should note that anxiety and depression levels observed in our sample of CTTH were low; therefore, it is possible that the interactions found in the current study maybe different in subjects with higher levels of anxiety or depression.

## Conclusions

This study found that a significant amount of variance in the relationship between headache frequency and the emotional burden of condition was indirectly mediated by depression and sleep quality, but not anxiety, in individuals with CTTH. Further, sleep quality also mediated the relationship between pain interference and the frequency of headaches. Our results would suggest that depressive symptoms and sleep quality play a relevant role in the chronicity of pain in individuals with CTTH. Future longitudinal studies will help to determine the clinical implications of these findings.

## Abbreviations

CFI: Comparative fin index
CTTH: Chronic tension type headache
GFI: Goodness of fit index
HADS: Hospital Anxiety and Depression Scale
HDI: Headache Disability Inventory
ICHD3: International Classification of Headache Disorders, third edition
NPRS: Numerical pain rate scale
PSQI: Pittsburgh Sleep Quality Index
RMSEA: Root mean square approximation
